# Investigation of Adverse Events Occurring during Rehabilitation in Acute Care Hospital

**DOI:** 10.3390/jcm11164706

**Published:** 2022-08-11

**Authors:** Tokio Kinoshita, Yukihide Nishimura, Yasunori Umemoto, Shinji Kawasaki, Shinnosuke Hori, Yoshinori Yasuoka, Motohiko Banno, Fumihiro Tajima

**Affiliations:** 1 Department of Rehabilitation Medicine, Wakayama Medical University, 811-1 Kimiidera, Wakayama 641-8509, Japan; 2 Division of Rehabilitation, Wakayama Medical University Hospital, 811-1 Kimiidera, Wakayama 641-8510, Japan; 3 Department of Rehabilitation Medicine, Iwate Medical University, 1-1-1 Idaidouri, Yahaba-cho, Shiwa-gun 028-3694, Japan

**Keywords:** accident, incident, occupational therapist, patient safety, physical therapist

## Abstract

Adverse events (AEs) during intensive care unit (ICU) rehabilitation and serious AEs during acute care hospital stays have been reported previously. However, no AEs have been reported for all patients needing rehabilitation in a non-ICU setting at an acute care hospital. This study aimed to investigate all AEs during acute-phase rehabilitation. Reports of AEs occurring during acute-phase rehabilitation in a university hospital from 1 April 2021 to 31 March 2022 were retrospectively analyzed. Minor and severe AEs were defined as those that did not require new treatment and those that required intensive treatment and/or prolonged hospitalization, respectively. There were 113 incidences of AEs during rehabilitation. The majority of AEs were minor (93.8%) and did not require new treatment. Only one serious AE was documented. The most common AEs were peripheral intravenous tube removal, decreased level of consciousness, poor mood due to low blood pressure, and falling down. There was no significant correlation between years of experience and the frequency of AEs. The neurosurgery department had the highest cases of AEs. Physical, occupational, and speech-language-hearing therapists had different characteristics and experiences of AEs. Risk management strategies should consider exercise load and targeted disorders due to differences in therapists’ specialties.

## 1. Introduction

The effectiveness of providing early rehabilitation for patients with many diseases has been demonstrated in recent years [1,2,3,4], and its benefits were observed for patients with coronavirus disease 2019 (COVID-19) [5]. Several guidelines and organizations recommend early mobilization from bed and exercise therapy at the onset of the disease [6,7,8]. However, early rehabilitation may be offered to patients with unstable respiratory and circulatory dynamics, or even to those on ventilators or various types of tubes [9,10], and thus may promote risks for adverse events (AEs) related to status changes and equipment failures. Therefore, practicing early rehabilitation also entails ensuring medical safety measures for patients.

It has been reported that most AEs occurring during rehabilitation in the intensive care unit (ICU) setting did not warrant additional treatment, cost, or prolonged hospitalization [11,12,13,14]. However, for patients in acute care hospitals, reports on AEs occurring during rehabilitative care are still lacking. Our medical hospital is an acute care hospital and a tertiary emergency care facility. Rehabilitation therapy is provided by a specialized team of physiatrists and registered therapists, depending on each patient’s condition [15,16]. We previously studied cases of AEs that occurred during rehabilitation, particularly those that had a serious impact on patients; results revealed that the occurrence of serious adverse events (SAEs) is not related to compliance with the criteria for discontinuation of rehabilitation or early initiation of rehabilitation [17]. However, in our previous report, only SAEs were targeted, and minor AEs remain to be explored.

The purpose of this study was to retrospectively evaluate all AEs that occurred during rehabilitation and to obtain detailed information to improve risk management strategies in future acute care settings.

Our study revealed that the majority of AEs that occur during rehabilitation in acute care settings have a minor impact on the patient, and the length of working experience of the therapist does not appear to affect the occurrence and frequency of AEs.

## 2. Materials and Methods

### 2.1. Study Setting

As of 2022, Wakayama Medical University hospital had 760 general beds (including 10 ICU beds) and 40 psychiatric beds, serving 27 clinical departments and 28 central medical treatment sections. Rehabilitation began upon request from the physicians in various departments to the department of rehabilitation.

Physiatrists examined inpatients prior to starting rehabilitation and evaluated their diagnosis, disease state, and physical condition. Registered and skilled therapists then commenced exercise therapy. A team meeting was held on the evening of the referral day of the patient to identify challenges and consider solutions, with the aim of improving rehabilitation safety and efficacy. For all patients participating in rehabilitation sessions, a physiatrist and therapist visited the patient’s room each morning to check vitals, consciousness, and any worsening of cognitive or motor impairments, and to assess the patient’s condition to determine if rehabilitation could continue. Therapists measured vital signs pre- and post-rehabilitation.

### 2.2. Study Design

In this retrospective cohort study, we analyzed AE reports that had been submitted by the Department of Rehabilitation Medicine to the Medical Safety Promotion Department at our hospital between April 2021 and March 2022. The AE reports were reviewed by the Risk Manager of the Rehabilitation Department and the Medical Safety Promotion Department, who also review the patient’s post-AE status and complaints. During the study period, one author (S.H.) worked as the department’s patient safety manager.

### 2.3. Data Collection Methods and Procedures

At our hospital, all staff are required to report an AE to their corresponding risk manager. An AE during rehabilitation means any instance that has caused or may have caused further physical or psychological injury to the patient. AEs include damage to the patient’s clothing and belongings. A decision is made by the Medical Safety Promotion Department concerning the impact of the AE level. According to the National Coordination Council for Medication Error Reporting and Prevention index [18], the impact of the AE is categorized into nine levels, as follows: category A (no error); categories B to D (error but no harm); categories E to H (error and harm); and category I (error and death). For AEs that occur in categories A to D, no additional treatment is required. Specifically, category B refers to an error occurring but not reaching the patient (an “error of omission” does reach the patient). Category C pertains to an error occurred that reaches the patient but does not cause harm. Category D is an error that reaches the patient and requires monitoring to confirm that it did not harm the patient and/or required intervention to preclude harm. For AEs that occur in category E, minor treatment is required. For AEs that occur in category F, intensive treatments are required, and/or an extension of hospital stay is needed. If permanent disability and sequelae with no significant or with significant functional or cosmetic problems develop, SAEs are defined as G or H, respectively; thus, SAEs are defined as categories F to I in reference to the above index.

The rehabilitation period was defined as the period when a therapist provided rehabilitation in a patient’s bedroom, corridor, training room, or outdoors. Specific definitions of the period include duration (1) from when a therapist enters a patient’s room until leaving, (2) from when a patient enters a training room until leaving, or (3) if a surveillance and/or assistance patient was moved with a transfer staff; duration was defined as the time from when a therapist received a patient to when a patient was handed over to a transfer staff member.

The survey items included the number of hospitalized patients, number of patients referred to the department of rehabilitation (inpatients), total number of AEs, degree of impact of AE on the patient, primary department of the patient who suffered from AE, content of the case, specialization of the in-charged therapist, and years of experience as a therapist.

### 2.4. Statistical Analyses

The values of the variables are given as numbers and means ± standard deviations (SD), where applicable. The hospital accepts residents in 2- to 6-month increments, and for residents, 12 months was calculated as 1. Thus, 6 months of residency was calculated as 0.5. Pearson was used to examine the correlation between years of experience and the number of AEs. Differences were considered statistically significant at *p*  <  0.05. Statistical evaluations were performed using the Graph Pad Prism 6 software (Graph Pad Software Inc., San Diego, CA, USA).

## 3. Results

The hospital’s division of rehabilitation had a total of 38 regular staff comprising 26 physical therapists, 8 occupational therapists, and 4 speech-language-hearing therapists. Our hospital accepts training for therapists who are licensed and already working in other facilities for studying in an acute care hospital. There were a total of 18 residents during the study period: 14physical therapists, 3occupational therapist, and 1 speech-language-hearing therapist. The number of years of experience was 10.1 ± 6.3 (mean ± SD) for regular staff. Table 1 shows the distribution of the years of experience of each therapist.

**Figure 1:**
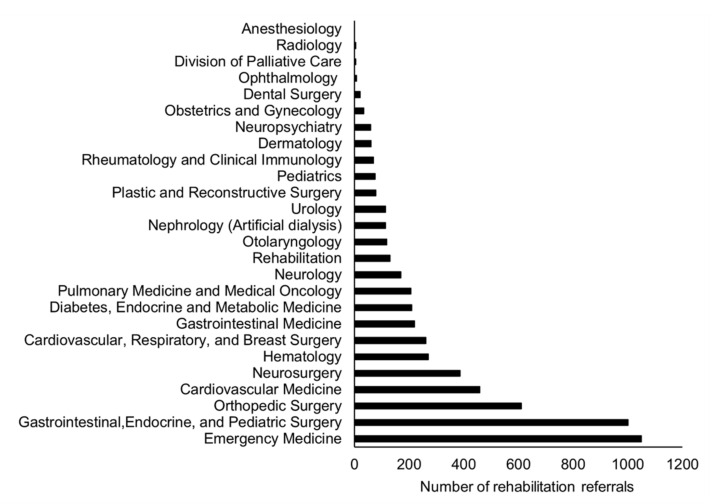
Annual number of patients referred to rehabilitation from each clinical department.

The total number of inpatients during the study period was 18,724, with an average length of stay of 12.2 days. There were 6945 patients referred to the department of rehabilitation, of whom 6400 and 545 were inpatients and outpatients, respectively. The most frequently referred clinical departments were Emergency Medicine (1050), Gastrointestinal, Endocrine, and Pediatric Surgery (1001), Orthopedic Surgery (611), Cardiovascular Medicine (458), Neurosurgery (386), Hematology (270), Cardiovascular, Respiratory, and Breast Surgery (261), Gastrointestinal Medicine (219), Diabetes, Endocrine and Metabolic Medicine (210), and Pulmonary Medicine and Medical Oncology (207) (Figure 1).

**Figure 2:**
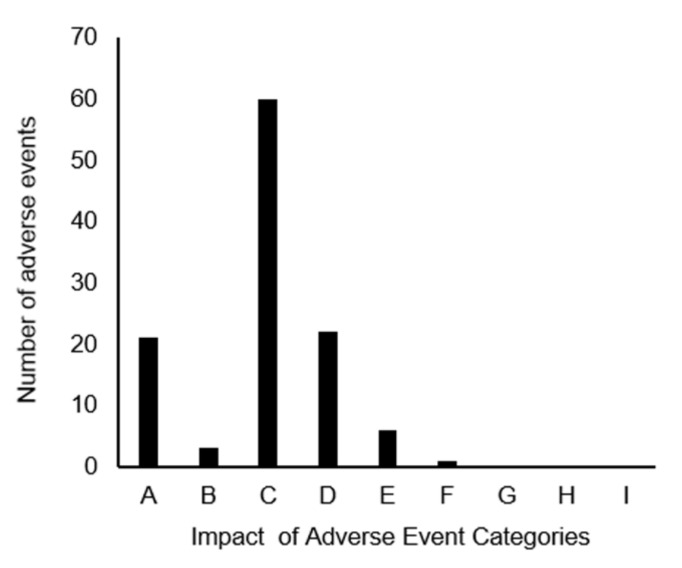
The degree of impact of adverse events on patients: category A (no error); categories B to D (error but no harm); categories E to H (error and harm): and category I (error and death), classified based on the National Coordination Council for Medication Error Reporting and Prevention index.

**Figure 3:**
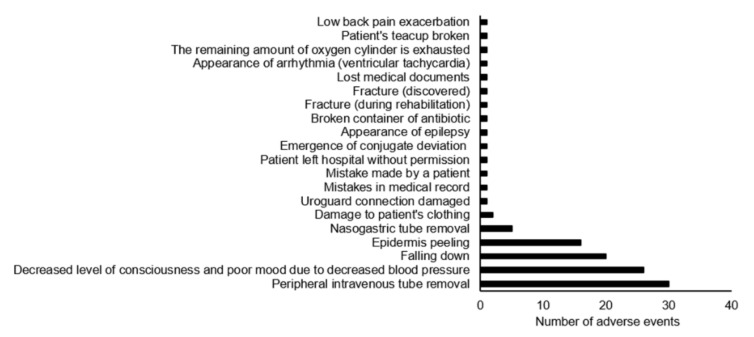
Content of adverse event cases and number of cases.

There were 3403 AEs (including 51 SAEs) in the entire hospital during the study period, and 113 (including 1 SAE) were recorded in the division of rehabilitation. In addition, all reports from the division of rehabilitation were on inpatients. The degree of impact of the AEs on the patients, as well as the corresponding contents and the number of occurrences, are shown in Figure 2 and Figure 3, respectively. The majority of AEs were minor events (93.8%) and did not require new treatment. There were six category E cases with minor treatment, which required treatment for epidermis peeling. Category F, classified as SAE, included one fracture during step climbing training during rehabilitation. AEs occurred in 94 rehabilitation rooms, 10 hospital wards, 7 ward corridors, and 2 other locations, with no reported incidents in the ICU. In addition, 30 events were recorded for peripheral intravenous tube removal, 26 for decreased level of consciousness and poor mood due to low blood pressure, 20 for falling down, and 16 for epidermis peeling.

**Figure 4:**
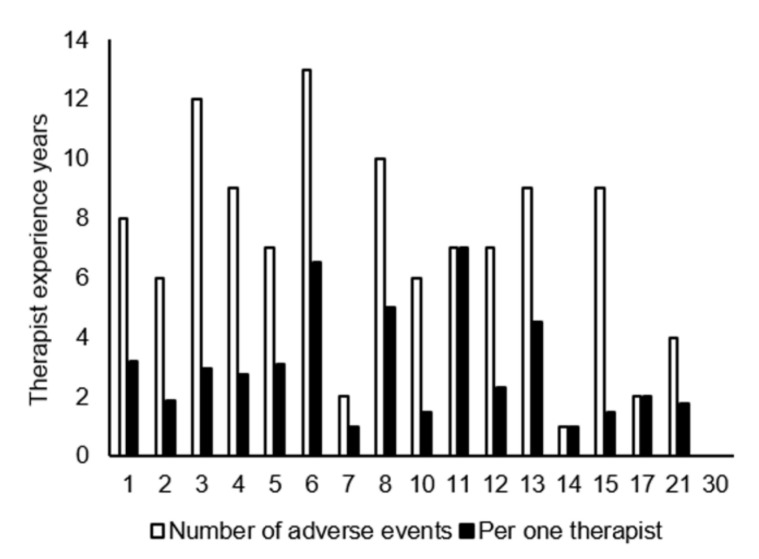
Number of adverse events by years of therapist experience. Per one therapist means the number of occurrences divided by the number of therapists enrolled in each year of experience. A clerical staff member was excluded for causing one adverse event.

**Figure 5:**
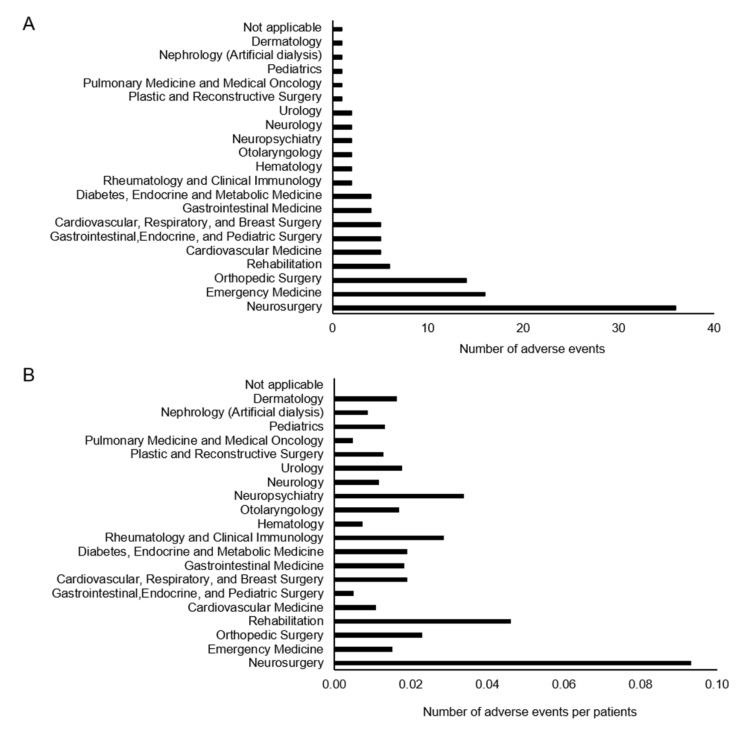
Number of adverse events by clinical department. (**A**) Number of adverse events by clinical department. (**B**) Number of adverse events by clinical department converted per patient. (**B**) results were obtained by dividing the number of adverse events by the number of people referred to the department of rehabilitation.

Figure 4 shows the number of occurrences of AEs relative to the years of experience of the therapist. Specifically, 6.5, 5, 7, and 4.5 average cases of AEs occurred during the therapist’s 6th, 8th, 11th, and 13th years of working experience, respectively. Meanwhile, AEs were less frequent during the first two years, with only one to three cases per year. Pearson’s results showed no significant correlation (r = −0.3857, *p* = 0.1263). The clinical departments with the most AEs recorded were Neurosurgery at 36 cases, Emergency Medicine at 16 cases, and Orthopedic Surgery at 14 cases (Figure 5A). Neurosurgery also had the greatest number of patients who suffered from AEs (Figure 5B).

Table 2 shows the number of AEs according to the specialization of the therapist. For physical therapists, the most frequent AE occurrences were decreased level of consciousness and poor mood due to low blood pressure in 22 cases, falling down in 17 cases, peripheral intravenous tube removal in 13 cases, and epidermis peeling in 10 cases. Meanwhile, occupational therapists had the highest number of AEs recorded for peripheral intravenous tube removal in 17 cases, epidermis peeling in 6 cases, and decreased level of consciousness and poor mood due to low blood pressure in 4 cases. The incidence of AEs related to peripheral intravenous tube removal was remarkably high, with each therapist having an average of 1.95 cases. Speech-language-hearing therapists did not have any AEs with a particularly high number of occurrences.

## 4. Discussion

The majority of AEs in this acute care hospital were minor events such as peripheral intravenous tube removal that did not require new treatment, and the length of working experience of the specialist did not contribute to such occurrence. Furthermore, the number of incidences was the highest in the Department of Neurosurgery, and the AE varies across therapy specializations. To the best of our knowledge, this study is the first to characterize AEs during rehabilitation in a non-ICU setting in an acute care hospital.

A systematic review with a meta-analysis conducted by Nydahl et al. reported 583 AEs in 22,351 physical exercise and mobilization sessions in the ICU, most of which were hemodynamic changes and hypoxic saturation [12]. The report noted that there was little impact on medical management or treatment caused by AEs, which could be resolved by sutures for falling, replacement following tube removal, increased oxygenation and end-expiratory positive pressure, and bed rest. SAEs were also not observed in a report investigating AEs when aggressive mobilization was performed in patients with intubated tracheas in the ICU [13].

Although the environment and patient conditions differ from previous ICU reports, it appears that SAEs are less likely to occur during inpatient rehabilitation in acute care hospitals outside of ICU settings. In this study, one SAE occurred in which a hip replacement patient suffered a fracture while climbing a step. The patient was able to walk with a cane 2 weeks after the surgery but had bone fragility resulting from a femoral fracture at the stem insertion site. We report that during the 7-year study period, only nine (0.32%) SAEs occurred during rehabilitation, all of which occurred in either the physical therapy room or the general ward [17]. Thus, SAEs can occur, albeit rarely, during rehabilitation in acute care hospitals.

A previous study on AEs in the ICU reported 34 cases occurring in 5276 sessions of rehabilitation, with 23 incidents of blood pressure instability or arrhythmia, 3 falls, and 2 tube problems [11]. Katsukawa et al. reported 13 AEs out of 198 mobilization sessions, of which 8 were in decreased blood pressure, 3 in heart rate instability, and no tube problems or falls. In addition, Lee et al., reported 26 AEs in 520 rehabilitation sessions, of which 11 cases were in respiratory distress, 6 in decreased oxygen saturation, 4 in tachypnea or bradycardia, and 1 in tracheal tube removal [14]. While the majority of AEs in ICUs are respiratory and circulatory, the most frequent AEs in this study were peripheral intravenous tube removal. None of the AEs in this study occurred in the ICU, suggesting that the different severity of the patient’s condition and medical management may have influenced the occurrence of AEs. When providing rehabilitation in an acute care setting, it is important to pay attention not only to respiratory and circulatory changes, but also to peripheral intravenous tube removal, falling down, and epidermal peeling.

In this study, the number of AEs was not particularly high among therapists with few years of working experience. In our previous report, there was no relationship between the occurrence of SAEs and the number of working years of the therapist in charge of the patient, indicating the need to be aware of the incidence regardless of experience [17]. In addition, neurosurgery patients had a higher incidence of AEs, with the most common AEs involving peripheral intravenous tube removal. Patients with cerebrovascular disease often suffer from symptoms that considerably reduce cognitive function and activities of daily living, including impaired consciousness, higher brain dysfunction, and motor paralysis [19]. In addition, the number of AEs may have been higher in the early stage of cerebrovascular disease because of the use of intravenous infusion through the peripheral intravenous tube [20]. Thus, patients with cerebrovascular disease may require special attention due to the possible occurrence of AEs.

The occurrence of AEs differed among physical therapists, occupational therapists, and speech-language-hearing therapists. Physical therapists perform loading methods, mainly gait and endurance training [21], and in Japan, they typically deal with the lower limbs and gait disorders. Meanwhile, occupational therapists deal with activities of daily living disorders [22], and in Japan, they mainly treat upper limb dysfunction. Speech-language-hearing therapists address dysphagia and communication disorders [23]. It was not surprising that the occurrence of AEs differs due to differences in primary symptoms and loading methods addressed by each therapist.

This study was limited in that it was a single-center, retrospective cohort study, and thus affected the generalizability of this study. Moreover, information concerning a non-adverse event group was not available. Consequently, it was not possible to analyze differences between patients without AEs. In the future, it is critical to conduct large-scale surveys across multiple facilities.

## 5. Conclusions

The majority of AEs that occur during rehabilitation in acute care settings have a minor impact on the patient, and the length of working experience of the therapist does not appear to affect the occurrence and frequency of AEs. In addition, peripheral intravenous tube removal and attention to patients with cerebrovascular disease are important to reduce AEs, as well as devising measures that consider exercise load and targeted disorders due to differences in therapists’ specialties.

## Figures and Tables

**Table 1:** Distribution of years of therapist experience among regular staff and residents.

Years of Therapist Experience	Number of Regular Staff (*n* = 38)	Number of Resident (*n* = 18) *	Total
1	1	1.50	2.5
2	2	1.25	3.25
3	3	1.08	4.08
4	3	0.25	3.25
5	2	0.25	2.25
6	2		2
7	2		2
8	2		2
10	4		4
11	1		1
12	3		3
13	2		2
14	1		1
15	6		6
17	1		1
21	2	0.25	2.25
30	1		1
Total	38	4.58	42.58

* The number of residents was 18, but the 12-month enrollment period is converted as 1; hence, the following conversions: converted to 0.17 for 2 months, 0.25 for 3 months, 0.33 for 4 months, and 0.5 for 6 months. Resident years of experience also include years of service at other facilities.

**Table 2:** Number of adverse events per physical therapist, occupational therapist, and speech-language-hearing therapist.

	Number of Occurrences	Per One Therapist *
**Physical therapist**		
Decreased level of consciousness and poor mood due to decreased blood pressure	22	0.75
Falling down	17	0.58
Peripheral intravenous tube removal	13	0.44
Epidermis peeling	10	0.34
Nasogastric tube removal	3	0.1
Uroguard connection damaged	1	0.03
Mistakes in medical record	1	0.03
Patient left hospital without permission	1	0.03
Emergence of conjugate deviation	1	0.03
Fracture (during rehabilitation)	1	0.03
Fracture (discovered)	1	0.03
Appearance of arrhythmia (ventricular tachycardia)	1	0.03
The remaining amount of oxygen cylinder is exhausted	1	0.03
Low back pain exacerbation	1	0.03
**Occupational therapist**		
Peripheral intravenous tube removal	17	1.95
Epidermis peeling	6	0.69
Decreased level of consciousness and poor mood due to decreased blood pressure	4	0.46
Falling down	2	0.23
Nasogastric tube removal	2	0.23
Damage to patient’s clothing	1	0.11
Appearance of epilepsy	1	0.11
Broken container of antibiotic	1	0.11
**Speech-language-hearing therapist**		
Lost medical documents	1	0.22
Damage to patient’s clothing	1	0.22
Falling down	1	0.22
Patient’s teacup broke	1	0.22
**Assistant staff**		
Mistake made by a patient	1	NA

NA, not applicable. * Number of adverse events divided by the number of therapists.

## Data Availability

The authors’ raw data supporting the conclusions of this article will be made available upon reasonable request.
